# The FBXL family of F-box proteins: variations on a theme

**DOI:** 10.1098/rsob.200319

**Published:** 2020-11-25

**Authors:** Bethany Mason, Heike Laman

**Affiliations:** Department of Pathology, University of Cambridge, Tennis Court Road, Cambridge CB2 1QP

**Keywords:** FBXL, ubiquitin, f-box protein, LRR, E3 ubiquitin ligases

## Abstract

The ubiquitin–proteasome system (UPS) is responsible for the rapid targeting of proteins for degradation at 26S proteasomes and requires the orchestrated action of E1, E2 and E3 enzymes in a well-defined cascade. F-box proteins (FBPs) are substrate-recruiting subunits of Skp1-cullin1-FBP (SCF)-type E3 ubiquitin ligases that determine which proteins are ubiquitinated. To date, around 70 FBPs have been identified in humans and can be subdivided into distinct families, based on the protein-recruiting domains they possess. The FBXL subfamily is defined by the presence of multiple leucine-rich repeat (LRR) protein-binding domains. But how the 22 FBPs of the FBXL family achieve their individual specificities, despite having highly similar structural domains to recruit their substrates, is not clear. Here, we review and explore the FBXL family members in detail highlighting their structural and functional similarities and differences and how they engage their substrates through their LRRs to adopt unique interactomes.

## 1. Ubiquitin signalling

Core cellular processes, like cell division and cell death induction, use protein degradation to bring about swift transitions and definitive outcomes for the cell. In eukaryotic cells, the ubiquitin–proteasome system (UPS) is a highly regulated and selective pathway controlling this process. The UPS specifies proteins for degradation by covalent conjugation of a 76 amino acid ubiquitin peptide which directs them to 26S proteasomes, for cleavage into short polypeptides and composite amino acids for reuse in the cell. The addition of ubiquitin onto proteins requires the orchestrated action of E1 (ubiquitin activating), E2 (ubiquitin conjugating) and E3 (ubiquitin ligating) enzymes in a well-defined ATP-dependent cascade [1–4]. Ubiquitin itself can be ubiquitinated on any of its seven internal lysines and one internal methionine. The resulting polyubiquitin chains vary in their topology resulting in a combinatorial complexity that allows for a multitude of functional outcomes [5,6]. The effects of some linkage types are well characterized, for example, K11 and K48 linkages are known to target proteins for proteasomal degradation and K48 linkages are the most abundant linkage type identified in organisms [7,8], while K63-linked chains have non-proteolytic functions such as activation and re-localization of proteins. The physiological consequences of the remaining ubiquitin chain linkages remain relatively uncharacterized despite their high abundance [5,9]. Recent advances in studying ubiquitin architecture have revealed that many ubiquitin chains are heterogeneous, consisting of multiple ubiquitin linkages in a single polymer or branched chains [10]. The discovery that ubiquitin can also be modified by ubiquitin-like modifiers SUMO and NEDD8, and more strikingly by phosphorylation and acetylation has increased the complexity of the ubiquitin code further [11]. Ubiquitin may therefore act as a signalling platform upon which more complex signals are assembled.

**Figure 1.**
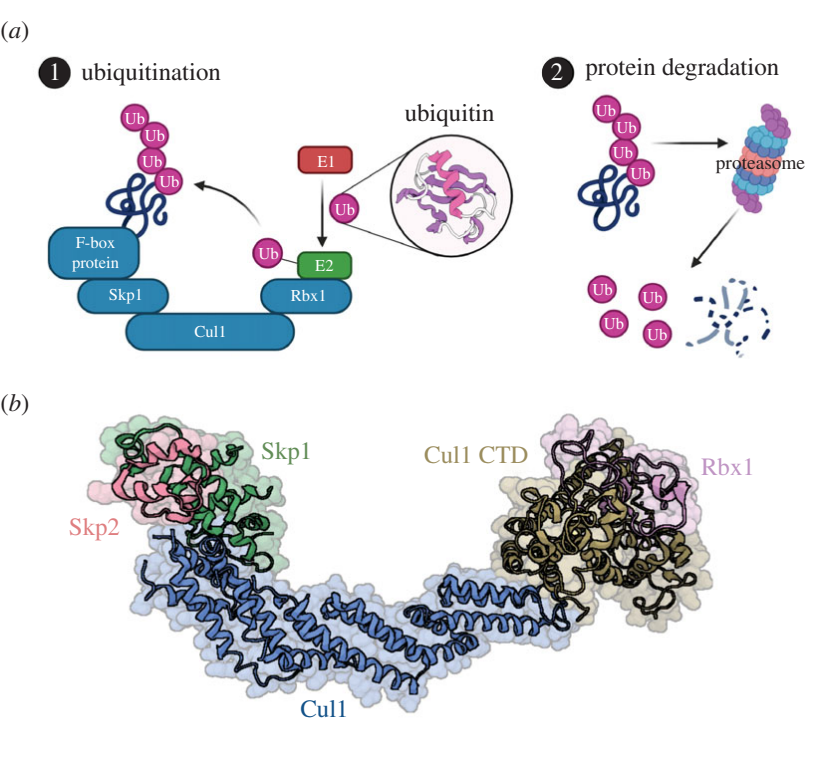
Schematic showing the SCF complex and ubiquitination of target proteins. Ubiquitination of target substrates requires the combined action of E1, E2 and E3 enzymes. (*a*) A schematic of the SCF E3 ubiquitin ligase in its characteristic ‘horseshoe’ conformation (blue). The scaffold protein Cul1 binds Rbx1 at its C-terminus and Skp1 at its N-terminus. Skp1 binds the substrate determining component, F-box protein, which recruits substrates to the complex. Rbx1 recruits the E2 ligase bound to ubiquitin and sequential ATP-dependent reactions transfer ubiquitin molecules to lysine residues on target substrates. Ubiquitinated proteins can then be directed to the proteasome for degradation (Ubiquitin PDB ID: 1UBQ [19]). (*b*) Three-dimensional structure of the SCF complex containing the FBP Skp2 (PDB ID: 1LDK. Rbx1 position inferred from PDB ID: 1LDJ, model is therefore a composite of both 1LDK and 1LDJ [20]). Model contains residues 15–55, 82–149, 154–216, 225–776 of Cul1; 19–106 of Rbx1; 2–37, 44–68, 84–140 of Skp1; and 109–149 of Skp2. Figure created with BioRender.com.

The diversity in ubiquitination signalling is achieved due to the wide range of enzymes that catalyse ubiquitination reactions [3,12–15]. Really Interesting New Gene (RING)-finger type E3 ubiquitin ligases comprise one of three classes of E3 enzymes that facilitate the transfer of ubiquitin directly from the E2 enzyme to the substrate [16]. The largest family of this type is the Cullin-RING E3 ligase (CRL) complex family, with over 200 members [17,18]. Within this family, the CRL1 or S phase kinase-associated protein 1 (Skp1)-Cullin 1-F-box protein (SCF) E3 ligases are the best characterized (figure 1). In these ligases, the CRL scaffold, Cullin 1 (Cul1), binds the Skp1 adaptor at its N-terminus and the RING-box protein Rbx1 at its C-terminus, thus bringing together two essential components required for E3 ligase activity. Skp1 recruits an F-box domain-containing protein (FBP), which acts as the substrate recognition component of the SCF ligase. Rbx1 engages an E2 enzyme conjugated to activated ubiquitin, thus enabling the direct transfer of ubiquitin to the substrate (figure 1). Skp1 binds to FBPs via their characteristic F-box domain (FBD), an approximately 50aa protein–protein interaction motif first identified in cyclin F (Fbxo1) and conserved in FBPs. The FBP : Skp1 dimer is a switchable unit that docks onto the cullin scaffold. Neddylation of Cullin1 (conjugation with the ubiquitin-like modifier Nedd8) activates the SCF complex causing a conformational change and increased ubiquitin ligase activity [21,22]. Following de-neddylation of Cul1, the FBP : Skp1 dimer is actively dissociated by the protein Cand1 (Cullin-associated NEDD8-dissociated protein 1) to regulate levels of active E3 ligases in the cell [23,24].

## 2. F-box proteins: receptors for (Skp1)-Cullin 1-F-box protein-type E3 ubiquitin ligases

In addition to containing an FBD for binding to Skp1 to engage the cullin scaffold, FBPs recruit substrates to the SCF ligase complex via variable protein–protein interaction domains, which allows for the recognition of a diverse range of substrates. The breadth of the variable protein-binding domain has led to a subclassifying nomenclature of the approximately 70 different FBPs: Fbxws contain WD40 repeats, Fbxls have leucine-rich repeats and Fbxos have other known binding domains such as proline-rich regions [25]. 42 FBPs have been shown to interact with Cul1 to form SCF-type E3 ubiquitin ligases complexes [24], but despite mounting evidence that FBPs have far-reaching cellular roles, concerted effort to date has focused on only a handful of FBPs, usually those associated with human diseases. While some redundancy in terms of substrate binding exists between FBPs from different subfamilies, in general each FBP has a unique repertoire of substrates and pathways that it regulates. But how the 22 FBPs of the FBXL family or the 8 FBPs of the FBXW family achieve their individual specificities, despite having similar structural domains to recruit their substrates, is not clear. The presumption is that a combination of the features we outline below for the FBXL family, the largest family of FBPs with a shared protein-binding domain, all likely play a role. These include variation in the number and sequences of binding domain repeats (in this case LRRs), the dynamic, responsive post-translational modification of degrons and the tissue-specific expression of FBPs, cofactors and substrates, which together determine a given E3 ligase's unique repertoire.

## 3. FBXL family: structural and phylogenetic relatedness of leucine-rich repeat family members

**Figure 2.**
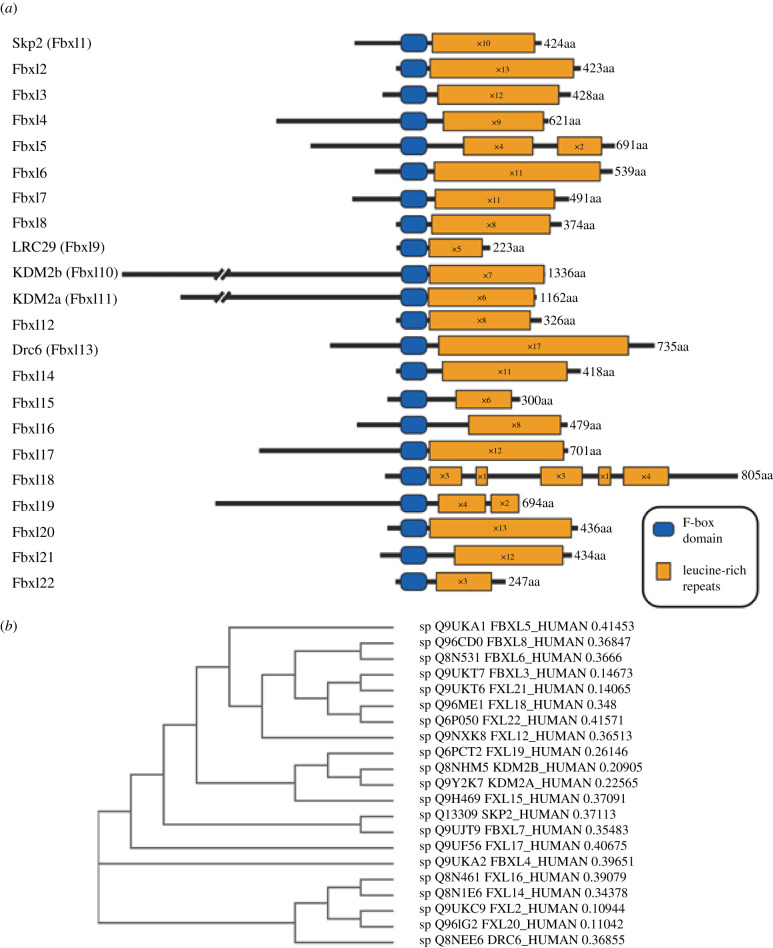
FBXL family members. (*a*) Schematic showing the F-box domains (blue) and LRRs (yellow) of 22 FBXL family members. The proteins are drawn to scale and centred around the FBDs. Number of LRRs as predicted by Robetta modelling and SMART predictions. (*b*) Phylogenetic tree depiction of relationships between FBXL F-box proteins. Generated using the ClustalW2 package (EMBL-EBI) with default settings (neighbour-joining tree without distance corrections). Distance values represent the number of substitutions (amino acid residues) as a proportion of the length of the alignment (excluding gaps).

**Figure 3.**
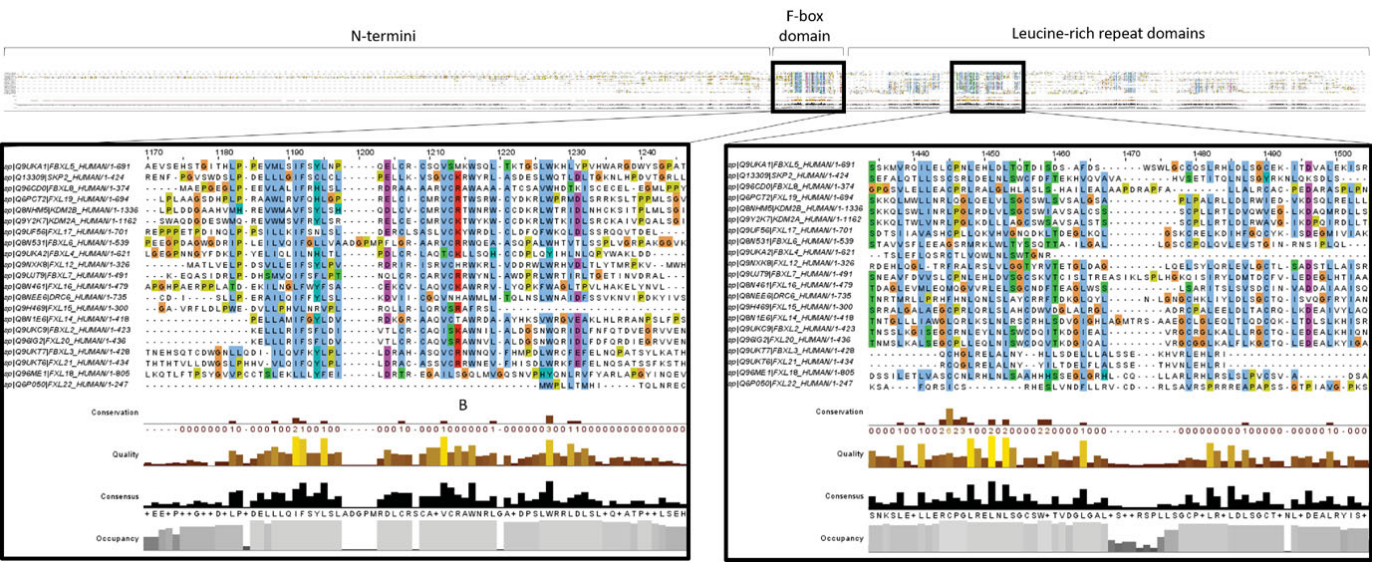
FBD and LRR multiple alignment. Multiple alignment of the FBXL protein sequences centred around the F-box domain region (left panel) determined by Clustal Omega (EMBL_EBI) and visualized using Jalview. ClustalX colouring of amino acids (blue greater than 60% hydrophobics (AILMFWVC); red greater than 60% positive charges (KR); magenta greater than 60% negative charges (ED); green greater than 50% polar (STQN); orange greater than 8% glycines (G); yellow greater than 8% prolines (P); cyan greater than 60% aromatics (HY). White amino acids are classed as unconserved. Multiple alignment centred around one example of a highly conserved region in the LRR domain (right panel).

The FBXL family represents the largest family of FBPs containing a common protein-recruiting domain. It typifies the diversity exhibited by FBPs with regard to substrate recognition, function and disease-association. The FBXL family of FBPs is composed of 21 members (Fbxl1–Fbxl8, Fbxl10–Fbxl22), each characterized by their leucine-rich repeat (LRR) domains. An additional potential member is leucine-rich repeat-containing protein 29 (LRC29), posited to be Fbxl9. All FBXL proteins contain an FBD and a variable number of C-terminal LRRs (figure 2*a*). Canonical LRRs are a repeating motif, 20–29 residues long that contain a conserved 11- or 12-residue consensus sequence (LxxLxLxxNxL or LxxLxLxxNxxL, where x can be any amino acid, L can be occupied by leucine, isoleucine, valine or phenylalanine (amino acids with hydrophobic side chains), and N is asparagine, cysteine, threonine or serine (amino acids with polar uncharged side chains)) [26,27]. In addition, Fbxl10 (KDM2B) and Fbxl11 (KDM2A) contain N-terminal JmjC domains, which are involved in the histone demethylation activity of these proteins, but this function will not be discussed further in this review [28]. In mammals, Fbxl20 is considered a paralogue for Fbxl2, as is Fbxl11 (KDM2A) for Fbxl10 (KDM2B), which is supported by their structural similarity and phylogenetic relatedness (figure 2). Multiple alignment of the FBXL protein sequences highlights the conserved nature of the FBD (figure 3, left panel), and also that the LRR domains are a highly ordered domain and very homologous between family members. In the expanded section in figure 3 (right panel), the alignment of the repeating leucine and other hydrophobic residues is evident across the majority of FBXL family members.

**Figure 4.**
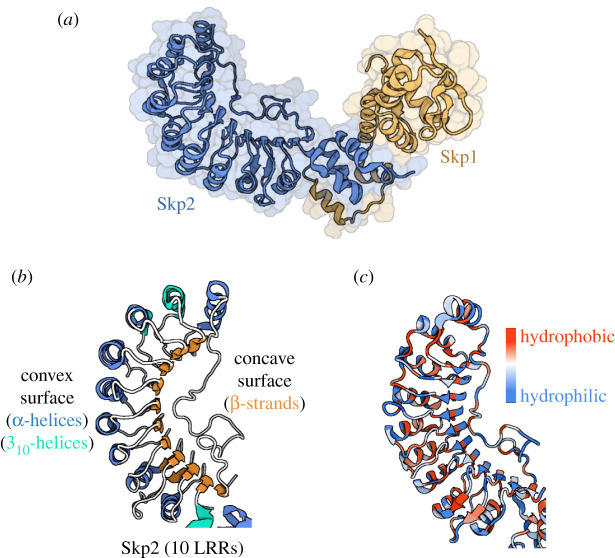
LRR structure of Skp2. (*a*) Crystal structure of the FBP Skp2 (blue) bound to the scaffold protein Skp1 (orange) (PDB ID: 1FQV [29]). (*b*) The three-dimensional structure of the leucine-rich repeat domain of Skp2, coloured according to secondary structure, blue, α-helices; turquoise, 3_10_-helices; orange, β-sheet (PDB ID: 1FQV). (*c*) As (*b*) but coloured according to hydrophobicity of residues. Red, hydrophobic; blue, hydrophilic. Figure created with BioRender.com.

Skp2 (Fbxl1), the FBXL founding family member, is the most extensively studied and best characterized FBP of this family. The crystal structure of Skp2 with its 10 LRRs was first solved in 2000, in complex with Skp1 [29]. The resulting structure revealed the curved solenoid shape adopted by LRRs (figure 4) [30]. The concave side of this ‘horseshoe’ is a series of parallel β-strands, and the convex side is composed of α-helices (figure 4*b*). The abundant leucine residues of LRRs form a hydrophobic core between the helices and sheets, resulting in a highly ordered structure (figure 4*c*). Skp1 binds to the F-box domain of Skp2 at a hydrophobic interface interdigitated with Skp1 and Skp2 structural elements (figure 4*a*). It is suggested that an analogous interface will form between most F-box protein family members and Skp1 to create a so-called core interface, which accounts for two thirds of the buried surface area [29].

**Figure 5.**
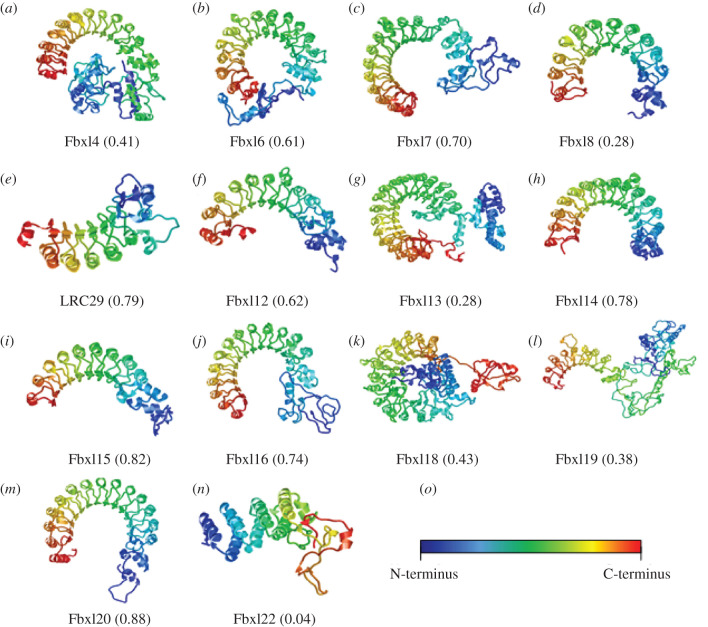
Three-dimensional structure prediction for FBXL family members. FBXL three-dimensional structure models generated by comparative modelling using Robetta protein structure prediction service. Numbers in brackets represent the prediction confidence value (scale of 0.0–1.0 where 1.0 is the highest confidence, confidence corresponds to the agreement in structure between the partial threaded models from the top alignment of each independent alignment method).

**Figure 6.**
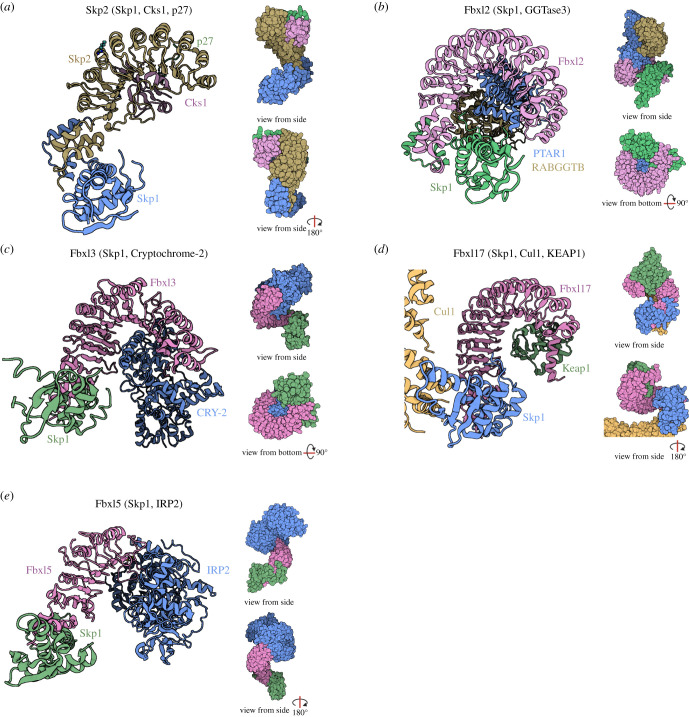
Three-dimensional structures of Fbxl proteins bound to substrates. (*a*) Crystal structure of the FBP Skp2 (Fbxl1) (brown) bound to Skp1 (blue) and the cofactor Cks1 (pink) in complex with a p27 peptide (green) (PDB ID: 2AST [33]). (*b*) Crystal structure of the FBP Fbxl2 (pink) bound to Skp1 (green) and GGTase3 (blue: PTAR1 and brown: RabGGTB) (PDB ID: 6O60 [31]). (*c*) Crystal structure of the FBP Fbxl3 (pink) bound to Skp1 (green) and the substrate Cryptochrome-2 (blue) (PDB ID: 4I6 J [32]). (*d*) Crystal structure of FBP Fbxl17 (residues 310–701, pink) bound to Skp1 (blue), Cullin1 (yellow) and the substrate KEAP1 (green) (PDB ID: 6WCQ [34]) (*e*) Crystal structure of FBP Fbxl5 (pink) bound to Skp1 (green) in complex with IRP2 (blue) (PDB ID: 6VCD [35]). Figure created with BioRender.com.

The structures of Fbxl2 (13 LRRs), Fbxl3 (12 LRRs), Fbxl5 (6 LRRs) and Fbxl17 (12 LRRs) have also been solved (figure 6) and show distinct curved LRR domains like Skp2 [31,32]. The three-dimensional structure of the remaining FBXL proteins have yet to be determined, so we used the Robetta protein structure prediction programme (http://new.robetta.org/) using comparative modelling to predict the entire three-dimensional structure of the remaining FBXL protein family members (excluding Fbxl10, 11 and 21) (figure 5).

As with Skp2, Fbxl2, Fbxl3, Fbxl5 and Fbxl17, the LRR regions of the other FBXL proteins are predicted to adopt a characteristic ‘horseshoe’ shape composed of α-helices and β-sheets. In general, the number of LRR repeats determines the degree of curvature adopted by the LRR region. Fbxl22, for example, has a relatively linear LRR region with only three predicted LRR repeats, compared to Fbxl13 which has 17 predicted LRRs and curves beyond 180° to form an almost circular LRR domain. Crystallization showed that the convex surface of Fbxl3 has a structural irregularity in LRR7/8 as a result of an extended β-strand, which disrupts the helical surface with an intra-repeat loop [32]. The Robetta-predicted models show that the canonical LRR pattern appears to vary slightly in other FBXL proteins too, resulting in loops extending or protruding from the ‘horseshoe’ (figure 5; see Fbxl12, Fbxl14 and Fbxl19). While the LRR domain is highly ordered and rigid in structure the relative position of the N-termini varies significantly in this modelling. This flexibility may enable the necessary conformational changes required to assemble an SCF complex, bind substrates and facilitate ubiquitination. Another possibility is that the compact structures represented by Fbxl4 and Fbxl18 may represent auto-inhibitory conformations adopted by FBXL proteins when not part of an SCF complex.

## 4. Substrate binding via leucine-rich repeats

The LRR domain is regarded as the substrate-recruiting domain for the E3 ligase. Crystal structures of four FBXL members (Skp2, Fbxl2, Fbxl3 and Fbxl17) in complex with binding partners have revealed that the concave surface of the LRR ‘horseshoe’ appears to be the preferred interface for protein binding [31–33]. Skp2 (Fbxl1) was the first FBXL protein to be studied by crystallography in complex with its substrate p27. However, Skp2 (Fbxl1)-mediated degradation of p27 requires the accessory protein Cks1 (CDK regulatory subunit 1). Analysis of the Skp2-Skp1-Cks1 ternary structure showed that Cks1 binds to the concave surface of the Skp2 LRR domain [33] (figure 6*a*).

Crystal structure analysis of Fbxl3 and one of its substrates further support this view. The Fbxl3-Skp1-mCRY2 complex is described as resembling an ice cream cone, with mammalian Cryptochrome 2 (mCRY2) sitting atop a base of Fbxl3-Skp1. Skp1 forms the very base of the cone and binds to the canonical 3-helix of the F-box domain in Fbxl3. The concave surface of Fbxl3's 12 LRRs then wraps up and around mCRY2, with the six most C-terminal LRRs in closest contact with the α-helical domain of mCRY2 (figure 6*c*) [32]. Mutations to residues in the LRR hydrophobic core or truncation of the C-terminus of Fbxl3 severely impaired mCRY2 binding [32].

Analogous to the ice cream cone shape adopted by Fbxl3 and mCRY2; the crystal structure of Fbxl2-Skp1-GGTase3 revealed an extensive, multivalent interface. Skp1 binds to the FBD of Fbxl2 forming an Fbxl2-Skp1 base which the GGTase3 prenyltransferase α-subunit PTAR1 binds (figure 6*b*). PTAR1 anchors itself onto the entire concave surface of the Fbxl2 LRRs with high affinity, likely attributed to the large intermolecular interface through which they interact. However, GGTase3 is not ubiquitinated by SCF^Fbxl2^, instead, Fbxl2 is the substrate for GGTase3, which geranylgeranylates it to alter Fbxl2 subcellular localization [31]. Thus, in addition to substrate recruitment, LRR domains also bind proteins that regulate the cellular E3 ubiquitin ligase repertoire.

Further mutational evidence has highlighted the importance of the concave surface of the LRR repeats for binding proteins. Crystal structure analysis of Fbxl17 bound to KEAP1 again shows complete encircling of the substrate by the LRRs, with residues in the last four LRRs directly engaging KEAP1 (figure 6*d*) [34]. A breast cancer-associated C627R mutation, predicted to be located within the concave surface of the LRR domain, in Fbxl17 prevents its binding to BTB-domain-containing proteins including KEAP1 [36]. Similarly, mutations that impaired binding of Fbxl3 to mCRY2 are localized on the concave surface of the LRR solenoid [32].

The interaction between Fbxl5 and iron regulatory protein 2 (IRP2) differs slightly as it does not require the concave surface of the LRRs. Fbxl5 captures IRP2 through its C-terminal end, with IRP2 bound at the distal end of the LRRs (figure 6*e*) [35]. No reports to date suggest that the convex surface of LRRs is involved in substrate recruitment, so it remains to be determined if this surface has other functions, especially since the protruding helices and loops revealed by the Robetta models introduce variability to this surface.

In addition to binding substrates, the LRRs have been shown to stabilize assembly of the SCF complex. The C-terminal tail of Skp2 extends back towards its FBD to insert at the interface between Skp1 and Skp2 [29], potentially strengthening their interaction. Consistent with this, truncating the C-terminal LRRs of Fbxl17 destabilizes the SCF ligase as demonstrated by a lack of SCF subunit recruitment and reduced ubiquitination activity [37]. This type of stabilizing interaction may only be possible for LRRs of a certain length. The Skp2/Fbxl1 and Fbxl17 LRRs are 10 and 12 repeats in length, respectively, so it is possible that family members with shorter repeats may not benefit from this effect and be more short-lived enzymes.

## 5. Substrate recognition by FBXL proteins: degrons, post-translational modifications and cofactors

The canonical model for FBP substrate engagement requires interactions via short degradation motifs (known as degrons) which are often primed by phosphorylation on specific serine or threonine residues prior to FBP binding (known as phosphodegrons) [38]. In the absence of phosphorylation, a protein is stable, but upon phosphorylation of the degron, the protein is ubiquitinated and degraded [39–41]. To increase stringency, some degrons can contain multiple phosphorylation sites or require the combined activity of multiple kinases before recognition by an FBP [4]. Other post-translational modifications (PTMs) can also be used in degrons, especially glycosylation and acetylation [38,42]. Some FBXL family members have been shown to use these degrons for recognition of proteins. For example, binding of Fbxl17 to Protein aRginine N-MethylTransferase 1 (PRMT1) requires the coordinated acetylation and deacetylation of lysine residues in a unique IKxxxIK motif [43]. In addition to PTMs, some FBPs employ the additional requirement for cofactors for specific target recruitment. As mentioned previously, Skp2 binding to p27 requires the accessory protein, Cks1, which recognizes phosphorylated Thr187 in p27 [33,44,45]. *In vitro,* the presence of Cks1 is required for the maximum ligase activity of SCF^Skp2^ towards phosphorylated p27.

Another example of cofactors employed by the Fbxl proteins is the utilization of the circadian repressor CRY2 as a cofactor for SCF^Fbxl3^ for its ubiquitination of c-Myc [46]. CRY2 and Fbxl3 form a heterodimer to cooperatively recruit c-Myc and promote its ubiquitination. Point mutations in CRY2 that disrupt its association with Fbxl3 prevent binding and subsequent ubiquitination of c-Myc. Both CRY1 and CRY2 are used for the recruitment of a second Fbxl3 substrate, Tousled-like kinase (TLK2), reinforcing the idea that Fbxl3 uses cofactors for substrate recruitment [47]. The use of such cofactors may fine-tune an Fbxl-mediated ubiquitination response to circadian rhythms or to cell cycle-linked events.

However, the requirement for cofactors is not absolute even among FBXL proteins that do use them. Other Skp2 substrates, like E2F1 and c-Myc for example, do not require Cks1 for ubiquitination. Moreover, not all FBXL proteins rely on PTM of their substrates in order to facilitate an interaction. Fbxl2 binds in a calcium-dependent manner to a calmodulin-binding IQ motif (I/LQXXXRGXXXR, LQERVDKVK) within phosphocholine cytidylyltransferase alpha (CCT*α*). The interaction between Fbxl2 and CCT*α* is sensitive to calmodulin, and entirely mediated through the IQ domain, since the mutation of a single residue within this IQ motif prevents Fbxl2 binding [48]. In some cases, PTMs can even inhibit substrate recruitment. Phosphorylation of Fbxl2 substrate p85*β*, a regulatory subunit of PI3K, inhibits their interaction. Fbxl2 rallies the tyrosine phosphatase PTPL1 to dephosphorylate Tyr655, which lies adjacent to the CaaX motif that mediates Fbxl2 binding. This then enables Fbxl2 binding and ubiquitination of p85*β* and its subsequent proteasome-mediated degradation [49]. The use of cofactors and PTMs, singly or in combination, can set thresholds for the sensitivity of a ubiquitin signalling response by the cell's E3 ligases.

## 6. Functional roles of FBXL proteins

Although the number of LRRs within members of the FBXL subfamily varies, the LRRs show clear similarities in terms of sequence homology, three-dimensional structure and substrate binding interfaces. Through the recognition and modification of substrates, FBXL proteins regulate multiple signalling pathways, many of which are therapeutically relevant disease-modifying pathways, and which are sometimes controlled by more than one FBXL protein. A recent review detailing the substrates for FBXL proteins has been published [50], but two dominant biological functions of FBXL family members are discussed here.

## 7. FBXL multi-faceted control of the cell cycle

Given the field-defining regulatory relationship between Skp2 and the cyclin-dependent kinase (CDK) inhibitor p27, the best-established functional role for an FBXL protein is in regulating the cell cycle. Skp2 recognizes p27 in a phosphorylation-dependent manner, with subsequent ubiquitination and degradation of p27 required for normal cell cycle progression [51]. The CDK inhibitors p21 and p57 are also substrates of Skp2 along with cyclin E, c-Myc and p130 [52–56]. However, other FBXL proteins also impact on cell cycle regulation via p27, including the degradation of calmodulin kinase I (CAMKI) by Fbxl12, which triggers G1 arrest by preventing CAMKI-mediated phosphorylation of p27 and assembly of the G1 phase kinase, Cyclin D1/Cdk4 [57]. Like Skp2, Fbxl12 also recruits p21 for ubiquitination, but this leads to increased levels of p21. Since SCF^Fbxl12^ assembles atypical ubiquitin chains containing both K48 and K63 linkages, these may help to maintain, rather than downregulate, the intracellular pools of p21 [58].

Other key cell cycle kinases regulated by FBXL proteins include the mitotic spindle regulator Aurora A kinase, which is ubiquitinated during mitosis by SCF^Fbxl7^ to promote its depletion. Overexpression of Fbxl7 leads to cell arrest and mitotic abnormalities, suggesting that the turnover of Aurora A by Fbxl7 must be finely tuned for the proper regulation of mitosis [59]. Another Aurora kinase family member, Aurora kinase B, is ubiquitinated by SCF^Fbxl2^ causing its degradation in the midbody. Fbxl2 also binds Cyclins D2 and D3, activators of the G1 phase Cdks, via a calmodulin-binding motif to promote their ubiquitination and degradation [60–62]. In addition, Fbxl2 regulates the transcription factor forkhead box M1 (FOXM1), the downstream targets of which include several cell cycle regulators [63]. Fbxl2 can be modified by O-GlcNAcylation, which suppresses the Fbxl2-mediated degradation of FOXM1 and contributes to gastric cancer pathogenesis [64]. Finally, although a major role for Fbxl3 is in the maintenance of the circadian clock oscillations, it also promotes the degradation of c-Myc, a ‘super-controller’ of cell proliferation, and of the kinase TLK2 [46,47]. More recently, it was shown that CRY1 and CRY2 cooperate with SCF^Fbxl3^ to regulate the E2F family of transcription factors [65], which are critical for the timely expression of cell cycle-regulated genes, hence revealing an intriguing connection between circadian clocks and cell cycle regulation. These examples highlight the multi-faceted interactions that FBXL proteins have during the cell cycle, ranging from direct regulation of G1 phase/mitosis transition kinases to cell cycle regulatory transcription factors, FOXM1, c-Myc and E2F family members.

## 8. FBXL coordination of the DNA damage response

Several FBXL family proteins participate in the DNA damage response, but as with the cell cycle, in opposing fashions. Fbxl5 negatively regulates human single-strand DNA binding protein 1 (hSSB1), a protein involved in the DNA damage checkpoints and recruitment of the MRN complex to double-strand breaks (DSBs) [66]. Overexpression of Fbxl5 sensitizes cells to genotoxic stress arising from impaired cellular responses to DSBs. In comparison, Fbxl12 is also involved in the response of cells to DSBs, but unlike Fbxl5, it acts to promote the DNA damage response. *Xenopus laevis* Fbxl12 is responsible for the ubiquitination and removal of the non-homologous end joining (NHEJ) initiating factor Ku80 [67]. Ku80, together with Ku70, binds DSBs and impedes repair by homologous recombination (HR). By promoting degradation of Ku80, Fbxl12 inhibits NHEJ and promotes HR. Finally, the phosphorylation of vacuolar protein-sorting 34 (Vps34) downstream of DNA damage-activated mitotic arrest, leads to its ubiquitination and degradation of Vps34 by SCF^Fbxl20^. Removal of Vps34 by Fbxl20-mediated ubiquitination leads to inhibition of autophagy. Thus, Fbxl20 engages a novel checkpoint for autophagy regulation as part of the DNA damage response [68]. FBXL proteins may therefore act to fine-tune the DDR mechanistically and interconnect it with other global cellular pathways.

## 9. FBXL roles in cancer

**Figure 7.**
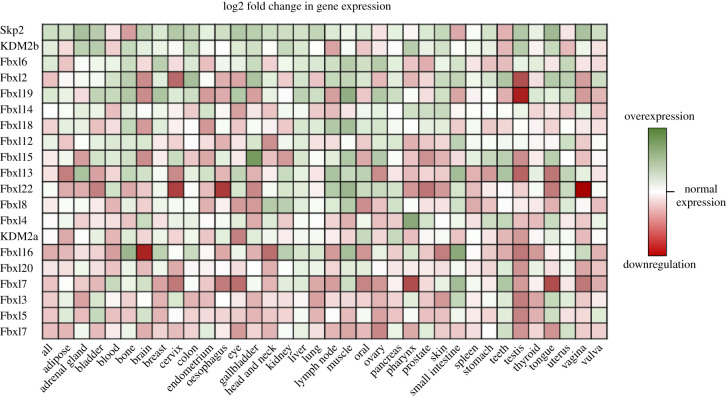
Gene expression profiles in normal versus tumour tissue for the FBXL family members. Heat map showing the Log2 fold change of gene expression of the FBXL family members in tumour tissue compared to normal tissue in the indicated tissue types. Data obtained from GENT2 (Gene Expression database of Normal and Tumour tissues).

Dysregulation in the control of the cell cycle and efficient DDR are key hallmarks of cancer, and as such, the FBXL proteins have often been shown to have a role in cancer pathogenesis [50]. Skp2 was identified as an oncogene because of its regulatory relationship with p27 and cell cycle regulation. Upregulation of Skp2 has been observed in many cancers, and high expression of Skp2 is often associated with poor prognosis. Several other FBXL family members have also been implicated in cancer, but unlike Skp2, are thought to act as tumour suppressors. We undertook a survey of FBXL gene expression levels in tumour tissues (figure 7). Consistent with its status as an oncogene, Skp2 shows the most widespread overexpression profile. Interestingly, the majority of FBXLs are predominantly downregulated in tumours and are thus likely to function as TSGs (figure 7). The most downregulated FBXL protein is Fbxl17 whose expression is decreased in almost all cancer types examined. In this light, it is worth considering the substrates of Fbxl17 in relation to how the loss of Fbxl17 may promote tumour development. Most of the Fbxl17 substrates identified to date are targeted for degradation following SCF^Fbxl17^-mediated ubiquitination. For example, in response to extrinsic oxidative stress, Fbxl17 promotes the rapid turnover of BACH1 to promote heme breakdown [69]. SCF^Fbxl17^ also ubiquitinates histone-modifying protein PRMT1, targeting it for proteasomal degradation [43]. By specifically recognizing inactive heterodimers of BTB-domain-containing proteins, Fbxl17 provides a quality control mechanism for dimer formation and can instruct aberrant dimers for degradation [36]. Lastly, Sufu (Suppressor of fused), a central regulator of Hedgehog (Hh) signalling, is degraded following ubiquitination by SCF^Fbxl17^ and exploitation of this leads to sustained Hh signalling in medulloblastoma [70]. However, in this instance, Fbxl17 is upregulated in a subtype of medulloblastoma tumours and thus functions as an oncogene. This highlights that FBXL proteins are not exclusively TSGs or OGs but can function as both in different contexts. Dysregulation of FBXL ligase activity as a result of FBXL loss or upregulation can both be contributing factors to cancer pathogenesis. This reinforces the need to uncover the ubiquitinome of FBXL proteins to fully understand the cellular pathways they control.

In contrast with these canonical UPS relationships, Fbxl17 was recently shown to bind Uap1, a key enzyme involved in the O-GlcNAcylation of proteins [37]. Fbxl17 binds Uap1 but does not promote its ubiquitination. Instead, Fbxl17 protects Uap1 from being phosphorylated, a modification that inhibits its activity. Thus, Fbxl17 maintains Uap1 activity by shielding it. Reducing Fbxl17 levels results in increased O-GlcNAcylation levels in cells, a phenomenon already reported in numerous cancer types and associated with poorer prognosis. Collectively these data show that although the loss of Fbxl17 in tumours can affect individual signalling pathways, it would also cause more widespread intracellular changes to protein homeostasis, heme metabolism and post-translational modifications. These more global destabilizing effects may explain the greater incidence of downregulated Fbxl17 expression in cancers more generally. The LRRs of Fbxl17 are important for binding substrates; however, they account for only half of the expressed protein. Fbxl17 along with other FBXL proteins, such as Fbxl4 and Fbxl19, also contain significant N-terminal portions that undoubtedly also contribute to the function and/or regulation of these proteins. In the case of Fbxl17, the ELM (Eukaryotic Linear Motif) resource predicts a globular domain from 92–175aa in the N-terminus that could be used to engage with further binding partners, and the ELM and PhosphoSitePlus databases predict several phospho-acceptor sites in the N-terminus, including for Cdks and GSK3*β* [71,72].

## 10. Tissue-specific expression of FBXL proteins

**Figure 8.**
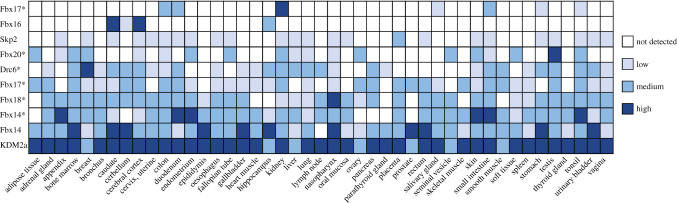
Protein expression levels in tissue. Protein expression levels of the indicated FBXL family members across 45 tissue types. Data obtained from the Human Protein Atlas, * indicates data classified as Uncertain-Inconsistency with, or lack of, RNA-seq and/or protein/gene characterization data, in combination with dissimilar staining pattern if independent antibodies are available.

Along with examining databases for the changes in gene expression of normal versus cancerous tissues, we surveyed the protein expression profiles of ten selected FBXL family members to gain some insight into which somatic tissues they may function in. FBXL proteins are expressed at low to medium levels across a wide range of tissues (figure 8). Of the 45 tissues surveyed, each expressed on average 6 FBXL proteins, with kidney and colon expressing 9 of the 10 FBXLs, while the prostate and parathyroid gland expressed only three out of 10 FBXLs tested. In considering individual FBXLs, KDM2A (Fbxl11) and Fbxl4 showed the highest expression in almost all tissue types surveyed. On the other hand, Fbxl16 had a surprisingly narrow range being expressed only in CNS tissues.

These data provide only a snapshot of FBXL proteins in somatic human tissues, and their expression is likely to be more dynamic and context-dependent, thus dictating their individual substrate repertoires. For example, Skp2, whose levels are known to oscillate with the cell cycle, shows maximum expression during G1/S phase, meaning its highest activity and range of substrates would be expected to be in the early part of the cell cycle of replicating cells, but not in quiescent cells. As FBXL proteins are implicated in a number of pathologies, including multiple different cancers and a mitochondrial DNA depletion syndrome [73], it may be possible to exploit their tissue-specific and responsive signalling capacity to modulate the disease-associated pathways they control [74].

## 11. Summary

FBXL family members bind to unique repertoires of proteins with far-reaching fundamental roles in the cell, some of which are already implicated in the pathogenesis of disease, and thus are potential targets for intervention. However, in order to therapeutically target FBXL family proteins and indeed FBPs in general, it is worth taking into consideration the characteristics described here, including the capacity for cross-talk among ubiquitin ligases, the use of different post-translationally activated or repressed degrons and cofactors to recognize their substrates, their varied tissue distribution and expression levels and their ability to ‘run interference’ in other signalling pathways. Recent efforts to co-opt the UPS with for example PROTAC technologies, to bring about targeted destruction, should take heed of the normal physiological ubiquitinomes for E3 ligases. Interference with these cellular pathways may have unintended consequences by precipitating pathological changes given the interlinked and contextual parameters of ubiquitin signalling.

## Data accessibility

This article has no additional data.
